# A Two-Step Strategy for Aroma Restoration of Strawberry Concentrate Based on ZIF-67@PDMS Composite Membrane

**DOI:** 10.3390/foods15020374

**Published:** 2026-01-20

**Authors:** Ziling Teng, Zixuan Ge, Xia Yu, Chunxia Zhou, Suling Guo, Yun Sun, Zhong Yao

**Affiliations:** 1 College of Food Science and Light Industry, Nanjing Tech University, Nanjing 211816, China; 18298771619@163.com (Z.T.); gezixuan0707@163.com (Z.G.); 18439480379@163.com (C.Z.); gsl8239@njtech.edu.cn (S.G.); sunyun_food@njtech.edu.cn (Y.S.); 2 Nanjing Zelang Biotechnology Co., Ltd., Nanjing 211816, China; roy@zl-pharm.com

**Keywords:** pervaporation, ZIF-67@PDMS composite membrane, strawberry juice, aromatic compounds, aroma restoration

## Abstract

An organophilic composite membrane, ZIF-67@PDMS, was fabricated to enhance the isolation of natural aromatic compounds. The as-prepared composite membrane was characterized using SEM, EDS, FTIR, XRD, and contact angle measurement. In comparison to pure PDMS, ZIF-67@PDMS, featuring a loading capacity of 2.5 wt% of PDMS and a membrane thickness of 15 μm, demonstrated markedly improved separation performance for the characteristic aroma compounds of strawberries, namely linalool, benzaldehyde, and ethyl acetate. Under optimal conditions, the permeation fluxes of the three compounds were 628.02 mg∙m^−2^∙h^−1^, 294.82 mg∙m^−2^∙h^−1^, and 254.14 mg∙m^−2^∙h^−1^, along with separation factors of 26.48, 7.94, and 6.32, respectively. ZIF-67@PDMS was then employed to isolate aromatic compounds from freshly squeezed strawberry juice. By backfilling the permeate, both the variety and the content of aromatic compounds in strawberry concentrate were notably restored, and its aroma profile also closely resembled that of fresh strawberry juice.

## 1. Introduction

The strawberry (*Fragaria* spp.), a perennial herbaceous plant from the *Rosaceae* family, is renowned for its fragrant, delectable, and vibrantly hued fruits. Moreover, strawberry fruits are abundant in functional nutrients such as vitamins, dietary fiber, and anthocyanins, which have been proven to offer health benefits, including antioxidant effects, vision protection, and digestive aid. Consequently, strawberries are considered one of the most significant economic crops globally, boasting an annual yield of over 10 million tons. To accommodate the substantial production capacity and reduce storage and transportation costs, fresh strawberries are frequently processed into concentrated juice through a series of procedures including juicing, filtering, concentration, and sterilization. This concentrated juice not only preserves the nutritional integrity and taste of the fresh strawberries but also adheres to stringent safety standards, thereby earning high favor among consumers. However, throughout the processing phase, especially during the concentration procedure, aromatic compounds in strawberries, such as esters, terpenes, furanones, and their derivatives [1], may undergo degradation (through hydrolysis or acetal reaction) or form azeotropic mixtures with water, leading to their loss. As a consequence, strawberry concentrate often draws criticism for its impaired flavor [2,3].

To tackle this problem, we proposed a two-step approach to restore the aroma of strawberry concentrate, which entails isolating volatile aromatic compounds from strawberry juice prior to its concentration, and then reintroducing them once the concentration process is finished, thus endowing the concentrate with the original aroma of the strawberry. In this two-stage approach, efficiently recovering aromatic compounds from strawberries is of vital importance. Unfortunately, owing to the extensive diversity and extremely low concentrations of aromatic compounds in strawberries [4], conventional separation techniques like solvent extraction, supercritical carbon dioxide extraction, and resin adsorption encounter considerable difficulties in terms of cost, poor thermal stability, solvent residues, and separation efficiency. This presents considerable challenges for practical application.

Pervaporation (PV) is a molecular-scale membrane separation technology that offers the merits of easy operation, low energy consumption, high selectivity, and exceptional safety. It has found extensive applications across diverse fields, such as chemical engineering, energy, environmental protection, pharmaceuticals, and fine chemicals [5,6]. Due to its solution-diffusion mechanism, PV technology is well-suited for separating near-boiling or azeotropic mixtures, as well as trace organic compounds from dilute solutions. Extensive research has demonstrated that membranes crafted from polydimethylsiloxane (PDMS), a type of silicone rubber material, are effective in isolating aromatic compounds from water [7]. Although aromatic compounds exhibit relatively low permeation flux owing to their large molecular size, high boiling points, and the limited free volume within the polydimethylsiloxane (PDMS) matrix, a high separation factor can still be attained [8,9]. This suggests that PV technology holds significant potential for application in the separation of natural aromatic compounds.

To further improve the separation performance of PDMS membranes, researchers have redirected their attention to the organic–inorganic composite membranes that are fabricated by incorporating porous nanoparticles like carbon nanotubes [10,11,12], metal–organic frameworks (MOFs) [13] or zeolite molecular sieves [14,15,16], into the PDMS matrix. Ali et al. [17] isolated pyridine from a pyridine–isooctane mixture using MIL-100 (Fe)/PDMS composite membranes, demonstrating a 2.2-fold increase in permeation flux compared to the original PDMS membrane. Li et al. [18] developed a multi-walled carbon tube (MWCNTs)/PDMS composite membrane for recovering methyl salicylate, linalool, and β-ionone from tea liquor, achieving a significant increase in permeation flux by 19.04%, 23.07%, and 50.00%, respectively, compared to its pure PDMS counterpart. By employing MCM-41/PDMS composite membranes, Hu et al. [8] achieved permeation fluxes of 0.95, 0.51, and 0.26 g∙m^−2^∙h^−1^ for 2-phenylethanol, β-ionone, and dihydroactinidiolide, respectively, in a water/aromatic compound system, demonstrating marked superiority over pure PDMS. The aforementioned studies revealed that the permeation flux of organic compounds could be effectively enhanced by incorporating porous nanoparticles into the PDMS matrix, thereby providing a feasible approach for the efficient recovery of aroma compounds from strawberry juice.

ZIF-67 is a type of metal–organic framework (MOF) material, constructed from cobalt ions (Co^2+^) linked with 2-methylimidazole ligands via coordination bonds, and featuring a zeolite-like sodalite topology [19,20]. Therefore, it possesses strong hydrophobicity and an exceptionally high specific surface area, which facilitates the adsorption of organic compounds [21]. Furthermore, ZIF-67 features a highly developed porous structure with a variety of pore sizes (0.34 and 1.1 nanometers), offering extra permeation pathways for permeates and thus holding promise for improving the permeation flux of aromatic compounds.

This study aims to address issues such as insufficient selectivity, flavor degradation, or solvent residues encountered during the processing of strawberry concentrate. A composite polydimethylsiloxane (ZIF-67@PDMS) membrane was fabricated using self-synthesized ZIF-67 crystals as the filler. An efficient, mild, and highly selective two-step strategy of “pervaporation–backfilling” was developed to recover and restore key characteristic aroma components in strawberry concentrate. Given food safety considerations, ethanol was chosen as the diluent instead of toxic *n*-heptane. The effects of particle loading, aromatic compound concentration, temperature, and membrane surface velocity on the separation performance of ZIF-67@PDMS membranes were investigated. Subsequently, the pervaporation (PV) process, utilizing a ZIF-67@PDMS membrane as the medium, was employed to recover aromatic compounds from freshly squeezed strawberry juice, and the post-backfilling sample was then subjected to SPME-GC/MS and electronic nose analysis to validate the feasibility of this aroma restoration approach, demonstrating potential for application to other fruit juices.

## 2. Materials and Methods

### 2.1. Reagents

Polydimethylsiloxane (PDMS) and dibutyltin dilaurate (DBTDL) were purchased from Shanghai Maclin Biochemical Technology Co., Ltd. (Shanghai, China); polyvinylidene fluoride (PVDF, 0.10 μm) membrane was purchased from Fujian Huamo Environmental Protection Co., Ltd. (Quanzhou, China); 2-Methylimidazole and ethyl acetate were purchased from Shanghai Merrell Biochemical Technology Co., Ltd. (Shanghai, China); cobalt nitrate hexahydrate was obtained from Xilong Science Co., Ltd. (Chengdu, China); tetraethyl orthosilicate (TEOS) was supplied by China National Pharmaceutical Group Chemical Reagent Co., Ltd. (Shanghai, China); linalool was obtained from Shanghai Yuanye Biotechnology Co., Ltd. (Shanghai, China); and benzaldehyde was obtained from Shanghai Aladdin Biochemical Technology Co., Ltd. (Shanghai, China). All other reagents were analytical grade.

### 2.2. Experimental Methods

#### 2.2.1. Preparation of ZIF-67 Nanoparticles

Cobalt nitrate hexahydrate and 2-methylimidazole with a molar ratio of 1:4 were dissolved in 200 mL of methanol. The 2-methylimidazole solution was added dropwise to the cobalt nitrate hexahydrate solution. The mixture was continuously stirred at room temperature for 1.0 h to yield purple crystals. After standing at room temperature for 24 h, the mixture was centrifuged, washed repeatedly with methanol, and dried overnight in a vacuum drying oven at 60 °C. The average particle size of the obtained ZIF-67 was about 400 nm.

#### 2.2.2. Preparation of ZIF-67@PDMS

A specific quantity of ZIF-67 particles was introduced into ethanol and subjected to ultrasonic dispersion for 1.0 h. The ultrasonicated ZIF-67 particle–ethanol mixture was employed as a diluent and blended with polydimethylsiloxane (PDMS) at a mass ratio of 1:18, followed by magnetic stirring for 30 min. Sequentially, tetraethyl orthosilicate (10 wt% of PDMS) and dibutyltin dilaurate (10 wt% of PDMS) were added, and the mixture was continuously stirred until it thickened to a viscosity of approximately 500–1000 cp. The viscous solution was poured onto a PVDF sheet and uniformly distributed with an adjustable coating apparatus, ensuring a film thickness of 15 μm. The prepared membrane was allowed to cure fully at room temperature, and then kept at 80 °C for 24 h. The pure PDMS membrane was fabricated following the aforementioned procedure, with ethanol employed as the diluent instead.

#### 2.2.3. Characterization of ZIF-67@PDMS

The surface morphologies and cross-section of ZIF-67@PDMS were observed using a scanning electron microscope (SEM, HITACHI S-4800, Suzhou, China). The duration of the gold spraying treatment was 90 s. The elemental composition of the samples was analyzed using energy dispersive spectroscopy (EDS, HITACHI S-4800, Suzhou, China) at a current of 10 μA and a voltage of 20 kV. The water contact angle (WCA) of the membrane material was measured using a fully automatic contact angle measuring instrument (OCA25, Raiffeisenstraße 34, 70794 Filderstadt, Germany). X-ray diffraction analysis (Mini Flex 600, Beijing, China) was performed on both ZIF-67 crystals and the composite membrane. The radiation source employed was Kα radiation excited by a copper target, with the scanning angle range configured from 5° to 55° and a scanning speed of 10° per minute. FT-IR analysis was carried out utilizing a Fourier transform infrared spectrometer (Nicolet 8700, Beijing, China). Potassium bromide pellets were prepared, and the scanning range was configured to span from 400 to 4000 cm^−1^.

#### 2.2.4. Pervaporation Experiment

The pervaporation experiments employed plate-type membrane modules with a membrane area of 25 cm^2^. The feed side temperature was controlled by a water bath, with the feed solution circulated via a peristaltic pump. Permeate was collected in a liquid nitrogen cold trap, while the permeate side pressure was maintained by a vacuum pump (approximately 400~600 Pa). The permeate flux (J) and the separation factor (α) were chosen as performance evaluation indices for the PV membrane.

The permeate flux (J) and separation factor (α) were defined as follows [22]:

(1) $$J\;=\;\frac{W}{A∆t}$$

where J represents the flux of the membrane material (g·m^−2^·h^−1^), W is the mass of permeate liquid (g), ∆t is the operating time (h), and A is the effective membrane area (m^2^).

(2) $$\alpha =\frac{Y_{A}/Y_{B}}{X_{A}/X_{B}}$$

where Y_A_ and Y_B_ are the mass concentrations of compounds A and B on the permeate side; and X_A_ and X_B_ denote the mass concentrations of compounds A and B in the retention solution, respectively.

#### 2.2.5. Flavor Restoration of Strawberry Concentrate

A 500 mL sample of fresh strawberry juice (provided by Nanjing Zelang Biotechnology Co., Ltd., Nanjing, China) was subjected to pervaporation separation for 9 h at 50 °C and with a feed rate of 0.32 L/min. A sample from the retentate side was taken and vacuum concentrated to 100 mL (water bath temperature of 60 °C). After the concentrated juice cooled, it was mixed with the permeate solution from the pervaporation process.

#### 2.2.6. Analytical Method

The concentrations of linalool and benzaldehyde were determined by high-performance liquid chromatography (HPLC, UltiMate3000). Sapphire C18 column (4.6 mm × 250 mm × 5 µm) was employed with mobile phase V (methanol)/V (water) = 75:25. Detection wavelengths were 220 nm (linalool) and 250 nm (benzaldehyde), with a flow rate of 0.8 mL/min, and a column temperature of 30 °C. The injection volume was 20 μL.

The concentration of ethyl acetate was determined using gas chromatography (Agilent 7890B, Beijing, China), which was equipped with a flame ionization detector (FID). Ethyl acetate was separated on an HP-5 (30 m × 320 µm × 0.25 µm) capillary column. The carrier gas (nitrogen) flow rate was 1.0 mL/min. The GC oven temperature was kept at 50 °C (5 min), then raised to 220 °C at 10 °C/min (3 min) and kept constant. The split ratio was set at 1:10, and the injection port temperature and the detector temperature were 220 °C and 300 °C.

Qualitative analysis of the aromatic compounds in strawberry juice employed the SPME-GC/MS method. The analytical procedure consisted of the following steps: 5.0 mL of sample solution was equilibrated in a water bath at 70 °C for 10 min. The 75 μm CAR/PDMS extraction head (57348-U) was inserted into the sample vial, positioned approximately 1 cm above the liquid surface, and stirring was conducted at 250 rpm/min during adsorption at 70 °C for 30 min. The extraction head was then removed and inserted into the GC-MS inlet for 10 min of thermal desorption, and the GC-MS was then run.

GC-MS detection conditions: A DB-5MS column (30 m × 0.25 mm × 0.25 μm) was used. The injection port temperature was 220 °C, and the split ratio was 1:10; helium carrier gas was added at a 1.0 mL/min flow rate. The ion source temperature was 230 °C, and the transmission line temperature was 280 °C. Programmed temperature ramp: 40 °C at the beginning for 3 min, then the temperature was increased at 3 °C/min to 100 °C, followed by a 10 °C/min ramp to 300 °C, and holding for 30 min; the MS scan range was 30~450 amu. The mass spectra of compounds were identified by matching with those in the NIST database and confirmed by retention indices (RIs). Quantitative analyses were performed by comparing the peak area of volatile compounds with the internal standard peak area, using 1,2-dichlorobenzene as an internal standard.

Electronic nose testing method: The conditions for determination included a sampling time of 1 s per group, a self-cleaning duration of 60 s for the sensor; the sample injection time was 5 s. The injection flow rate was 400 mL/min. The analysis sampling time was 80 s, and each sample group was tested in parallel three times. Data was taken from the 69–71 s interval.

#### 2.2.7. Data Processing

All the samples were tested three times in parallel, and the results are presented as mean ± standard deviation. Data points in figures and tables represent the mean of the three independent replicates, with error bars denoting the standard deviation. Qualitative analysis of volatile components in the samples was performed using GC-MS Solution 4.50C software, the NIST database, and retention indices (RIs). Electronic nose data were processed and analyzed with WinMuster V2.x software. All experimental data presented herein represent the mean values of three parallel experiments.

## 3. Results and Discussion

### 3.1. Effect of Particle Packing Amount on Separation Performance

**Figure 1:**
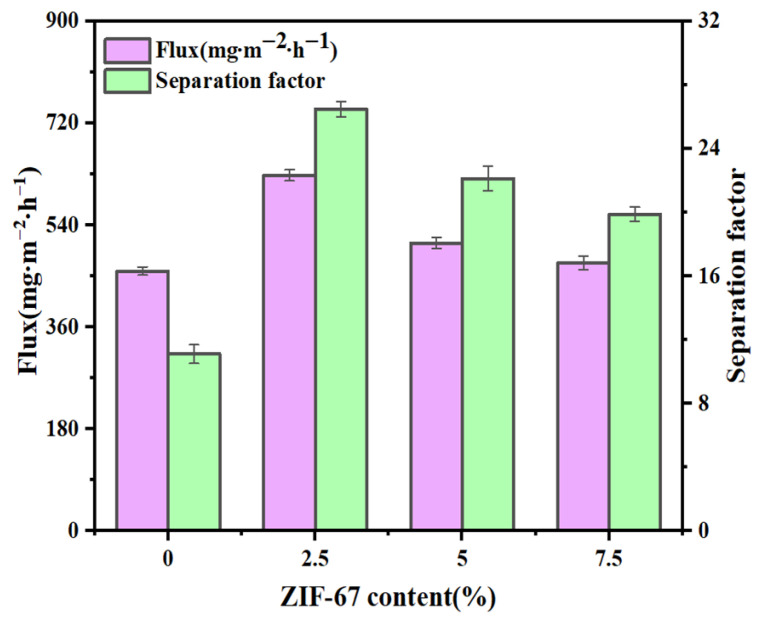
Effect of ZIF-67 dosage on linalool permeation flux and separation factor.

The separation performance of ZIF-67@PDMS with ZIF-67 loading levels of 0%, 2.5%, 5%, and 7.5% was investigated in a linalool–water system. As shown in Figure 1, the separation performance of ZIF-67@PDMS was optimal at a particle loading of 2.5%, with a separation factor and permeation flux of 26.48 and 628.02 mg·m^−2^·h^−1^, respectively. This represented a significant 36.83% improvement over the pure PDMS membrane, likely attributable to the unique nanoscale dual-pore structure of ZIF-67 providing additional pathways for linalool permeation and diffusion [23]. However, the separation performance of ZIF-67@PDMS gradually declined with increasing particle loading. This phenomenon may stem from the fact that, as the loading of nanoparticles increases, their growth mode shifts from single-layer dispersion to three-dimensional aggregation. A large number of crystal nuclei grow and fuse with each other under spatial constraints, forming aggregates with rich interparticle interfaces, resulting in higher surface energy and reduced free volume within the separation layer. The formation of internal interfaces in such aggregates can block pore channels and introduce surface defects such as coordination-unsaturated metal sites [24]. These changes collectively hinder the permeation and diffusion of linalool. Nevertheless, such structural alterations exert minimal influence on water molecule diffusion, consequently diminishing the separation selectivity of the ZIF-67@PDMS membrane.

### 3.2. Characterization of the ZIF-67@PDMS Composite Membrane

**Figure 2:**
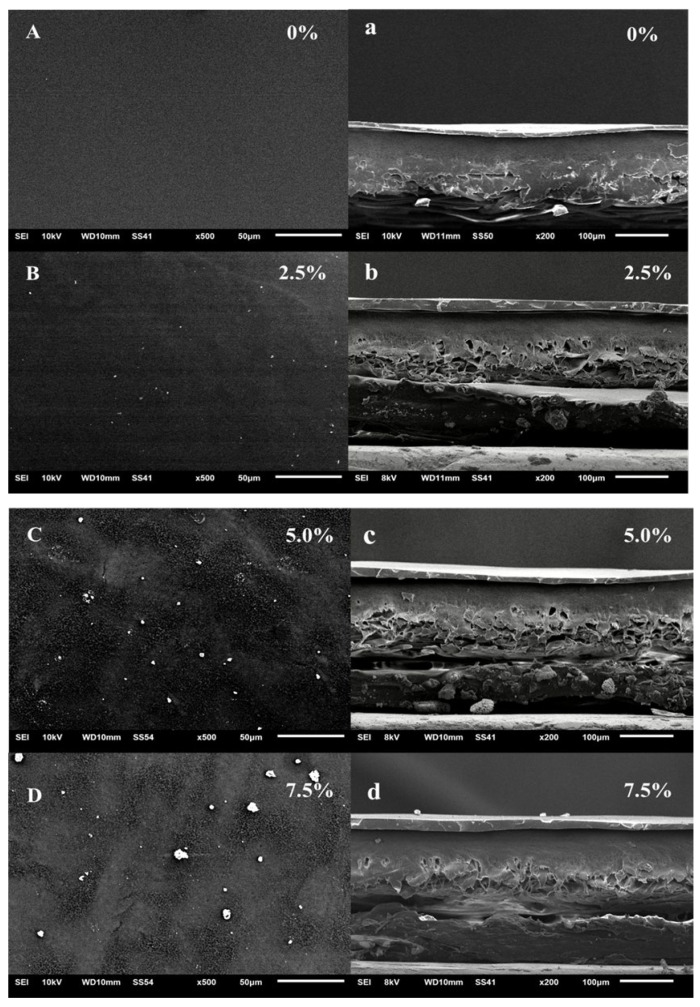
Surface (**A**–**D**) and cross-section (**a**–**d**) morphology of ZIF-67@PDMS membrane.

**Figure 3:**
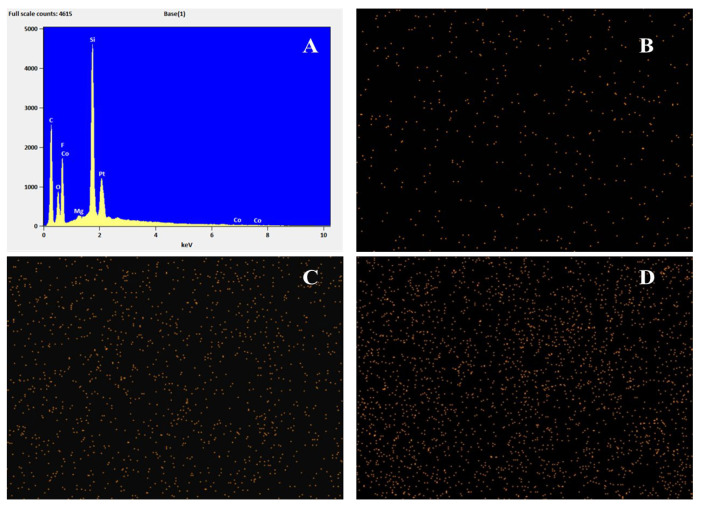
Mapping of ZIF-67@PDMS films ((**A**) Elemental Mapping; (**B**) 2.5 wt%; (**C**) 5.0 wt%; (**D**) 7.5 wt%).

The surface and cross-sectional morphology of the PDMS membrane and the synthesized ZIF-67@PDMS hybrid membrane were observed using the SEM-4800 scanning electron microscope. The pure PDMS membrane was smooth with no apparent defects, and the separation layer was dense and uniform (Figure 2A,a). Composite membranes with varying ZIF-67 doping levels exhibited varying degrees of particle agglomeration on their surfaces that became more pronounced with increasing filler loading. This phenomenon resulted in both enlarged surface particles and the formation of encapsulated aggregates within the separation layer, significantly compromising its structural uniformity and integrity. Further EDS analysis indicated that the Co content in ZIF67@PDMSmembranes increases with loading and remains evenly distributed (Figure 3). As shown in Figure 3A, cobalt (Co), indicative of the characteristic metal center in ZIF-67, was uniformly distributed throughout the membrane. This observation directly demonstrated the successful doping and good dispersion of ZIF-67 particles within the PDMS matrix, aligning with the SEM results. Silicon (Si) and oxygen (O), the main constituent elements of the PDMS polymer backbone (-Si-O-Si-), exhibited widespread distribution, confirming the presence of a continuous PDMS phase. Fluorine (F) primarily originated from the polyvinylidene fluoride (PVDF) porous support membrane. Additionally, the detected platinum (Pt) was attributed to the gold (Au)/platinum (Pt) coating applied during SEM sample preparation to enhance conductivity. As an extrinsic element not inherent to the membrane, its distribution also indirectly reflected the surface morphology of the film. The signal for magnesium (Mg) was weak, possibly stemming from environmental or instrumental background during the experiment. Collectively, the elemental mapping results indicated that ZIF-67 particles were successfully and uniformly incorporated into the PDMS separation layer.

**Figure 4:**
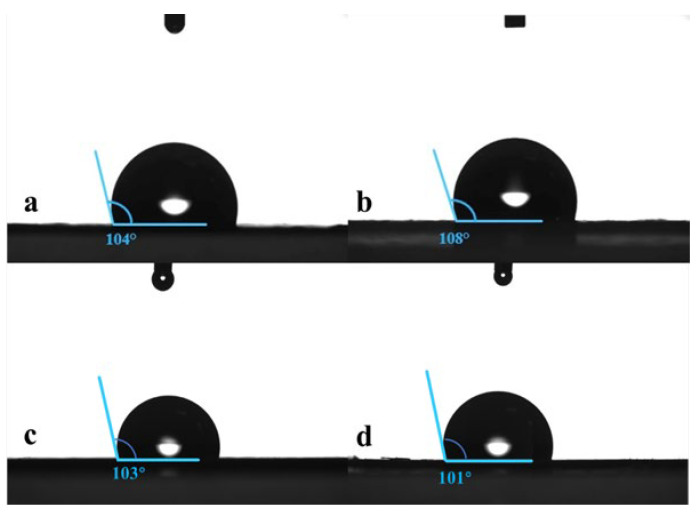
Water contact angle analysis of ZIF-67@PDMS membranes. ZIF-67 content: (**a**) 0 wt%; (**b**) 2.5 wt%; (**c**) 5.0 wt%; and (**d**) 7.5 wt%.

The water contact angle measurements for the ZIF-67@PDMS membrane are shown in Figure 4. The water contact angle of the pure PDMS membrane was 104°, exhibiting strong hydrophobicity; the surface water contact angle of the ZIF-67@PDMS membrane was between 101° and 108°, similar to that of pure PDMS, indicating that the incorporation of ZIF-67 particles had no significant effect on the hydrophobicity of the composite membrane.

**Figure 5:**
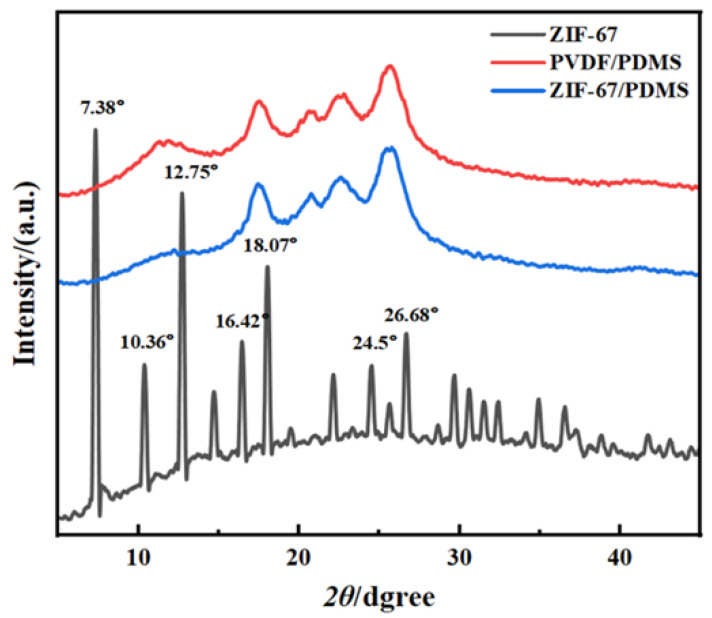
XRD patterns of ZIF-67 particles, PDMS membrane, and ZIF-67@PDMS composite molds.

XRD analysis revealed distinct diffraction peaks for ZIF-67 particles at 2θ angles of 7.38°, 10.36°, 12.75°, 16.42°, 18.07°, 24.50°, and 26.68° (Figure 5). Among these, the diffraction peaks at 7.38°, 10.36°, and 12.75° are characteristic of ZIF-67 [25], corresponding to its (110), (200), and (211) crystal planes, respectively, and demonstrated the integrity of the particle structure. The XRD patterns of both the pure PDMS membrane and ZIF-67@PDMS did not exhibit sharp and intense diffraction peaks. This was attributed to the encapsulation of ZIF-67 particles within the PDMS polymer matrix, resulting in an amorphous character [26].

**Figure 6:**
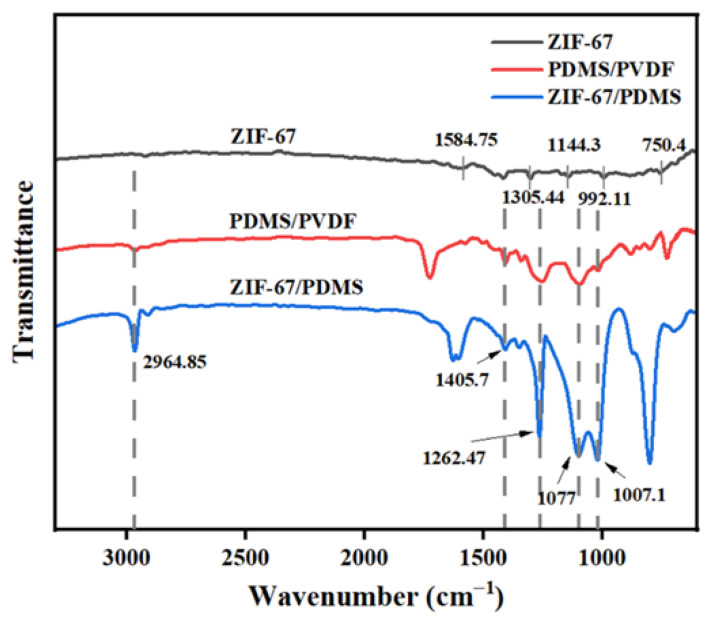
FTIR-ART spectra of ZIF-67 particles, PDMS membrane, and ZIF-67@PDMS composite molds. Dashed lines: visual guide lines.

Figure 6 presents the FTIR spectra of ZIF-67 particles, pure PDMS, and the ZIF-67@PDMS composite membrane. In the ZIF-67 spectrum, a characteristic vibration peak for the Co-N bond appears at 750 cm^−1^, and characteristic peaks at 1584 cm^−1^, 1144 cm^−1^, and 992 cm^−1^ are attributed to the asymmetric and symmetric stretching of the conjugated C=N and C-N bonds in the heterocycle. The peak at 1305 cm^−1^ arises from structural vibrations of the imidazole ring skeleton, and these vibrational modes are closely related to the aromatic characteristics and hydrogen bonding interactions of the imidazole ring system, confirming ligand coordination to the metal center [27]. For the PDMS membrane, the characteristic peak at 2964 cm^−1^ is attributed to the asymmetric and symmetric stretching vibrations of -CH [28]; the absorption peaks at 1405 cm^−1^ and 1263 cm^−1^ correspond to the deformation vibrations of Si-CH, while the peaks at 1077 cm^−1^ and 1007 cm^−1^ represent the asymmetric stretching vibrations of Si-O-Si in the PDMS backbone [9]. Compared to the PDMS membrane, the ZIF-67@PDMS composite membrane did not exhibit new absorption peaks, indicating that PDMS and ZIF-67 particles were only physically blended without forming new chemical bonds.

### 3.3. Separation Performance of ZIF-67@PDMS Composite Membrane for Linalool, Benzaldehyde, and Ethyl Acetate

#### 3.3.1. The Effect of Aromatic Compound Concentration

**Figure 7:**
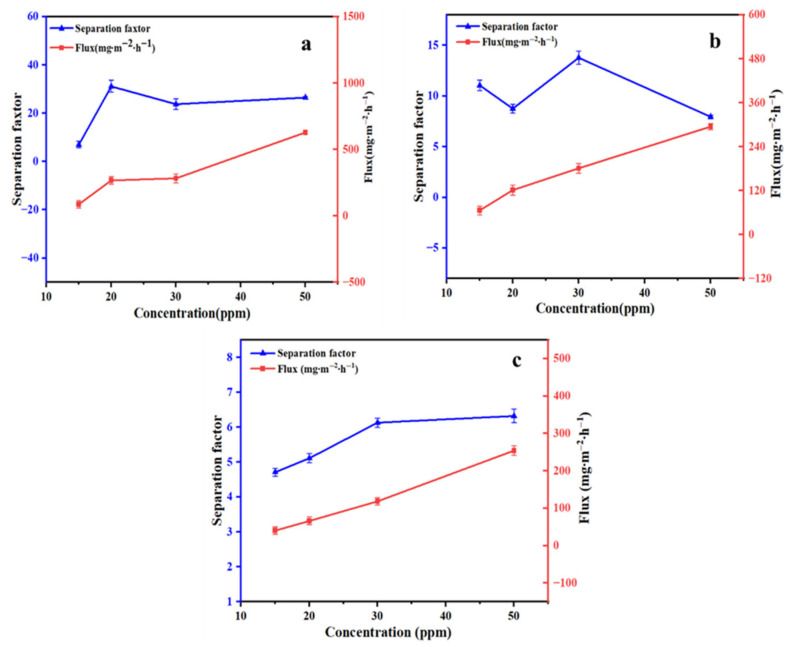
Pervaporation performance of the ZIF-67@PDMS composite membrane for linalool (**a**), benzaldehyde (**b**), and ethyl acetate (**c**) at different concentrations.

The effects of feed concentration on the separation performance of the ZIF-67@PDMS membrane were studied. Aqueous solutions of linalool, benzaldehyde, and ethyl acetate with concentrations (15.0 mg/L to 50.0 mg/L) were prepared and subjected to pervaporation separation for 2 h at 50 °C and a flow rate of 0.32 L/min. As shown in Figure 7, the flux of all three of the aromatic compounds increased with feed concentrations. At a feed concentration of 50 mg/L, the permeation fluxes of linalool, benzaldehyde, and ethyl acetate reached their maximum values, which were 628.02 mg·m^−2^·h^−1^, 294.82 mg·m^−2^·h^−1^, and 254.14 mg·m^−2^·h^−1^, respectively, with separation factors of 26.48, 7.94, and 6.32. This is because the higher concentration of aromatic compounds increases adsorption in the membrane, enhancing the driving force for permeation [29]. Meanwhile, increased organic concentration in the feed solution promoted their sorption onto the PDMS layer and induced membrane swelling. This weakened the interaction forces between polymer chains, increased the free volume of the membrane, and thereby enhanced the diffusion and overall permeation flux of organics [30].

#### 3.3.2. The Effect of Temperature

**Figure 8:**
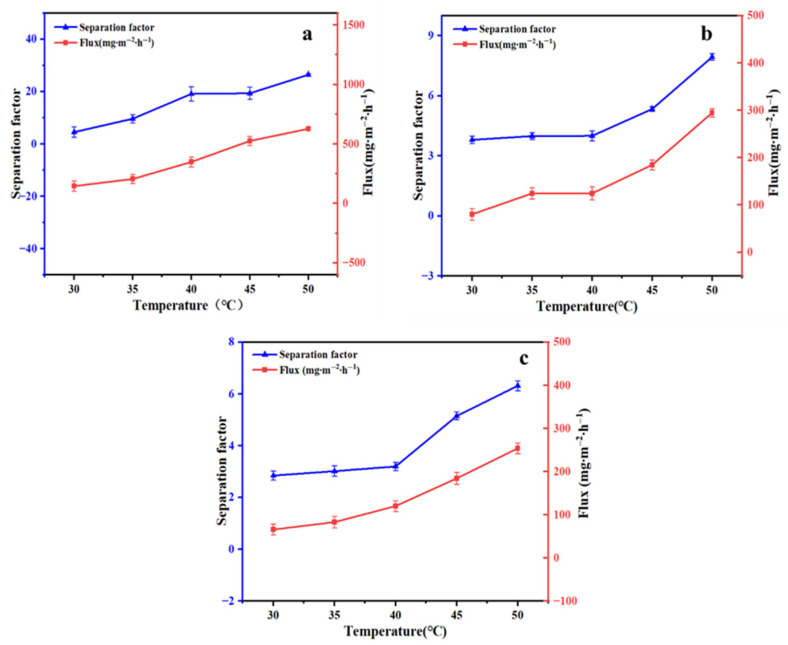
Pervaporation performance of the ZIF-67@PDMS composite membranes for linalool (**a**), benzaldehyde (**b**), and ethyl acetate (**c**) at different temperatures.

The effect of operating temperature on membrane performance was also explored. Aqueous solutions of 50 mg/L of linalool, benzaldehyde, and ethyl acetate were prepared and subjected to pervaporation separation for 2.0 h at a flow rate of 0.32 L/min and temperatures of 30 °C, 35 °C, 40 °C, 45 °C, and 50 °C. As shown in Figure 8, the flux and separation factor of the three aromatic compounds increased as the operating temperature increased. This might be attributed to several factors: first, the increase in temperature made the polymer molecular chains more active, expanded the free volume within the membrane, and provided more channels for mass transport [31]. At the same time, due to the intensified molecular thermal motion, the solute diffusion rate increased significantly as the temperature rose. ZIF-67 particles played a dual role in the mixed matrix pervaporation membrane: on the one hand, their spatial confinement effect suppressed excessive polymer chain swelling, effectively controlled the rise in water flux, and improved the aromatic separation factor; on the other hand, physical cross-linking by the particles reinforced the membrane’s structural stability, markedly improving both permeation flux and separation selectivity [32].

The relationship between temperature and flux follows the Arrhenius equation [33]:

(3) $$J\;=\;J_{0}\exp(\frac{{-E}_{J}}{\mathrm{RT}})$$

where J, J_0_, EJ, R, and T denote flux (mg·m^−2^·h^−1^), pre-exponential factor, apparent activation energy (kJ/mol), gas constant (8.314 J·mol^−1^·K^−1^), and feed temperature (K), respectively.

**Figure 9:**
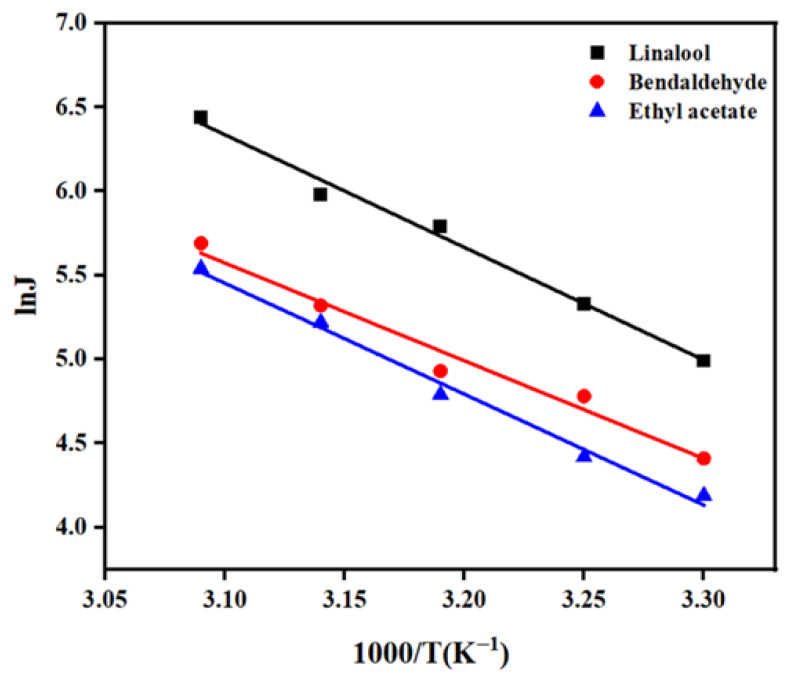
Fitting plots of permeation activation energy for linalool, benzaldehyde, and ethyl acetate.

The fitted results activation energies for the three aromatics are shown in Figure 9. The activation energies for linalool, benzaldehyde, and ethyl acetate are 55.71 kJ/mol, 48.38 kJ/mol, and 54.86 kJ/mol, respectively. All activation energy values were positive, indicating that an increase in feed temperature led to an increase in flux. Linalool exhibited a slightly higher activation energy, indicating that it was more sensitive to temperature changes and showed a more pronounced increase in flux with rising temperature [34]. This might be related to its relatively low boiling point (198 °C) [35].

#### 3.3.3. The Effect of Membrane Surface Flow Rate

**Figure 10:**
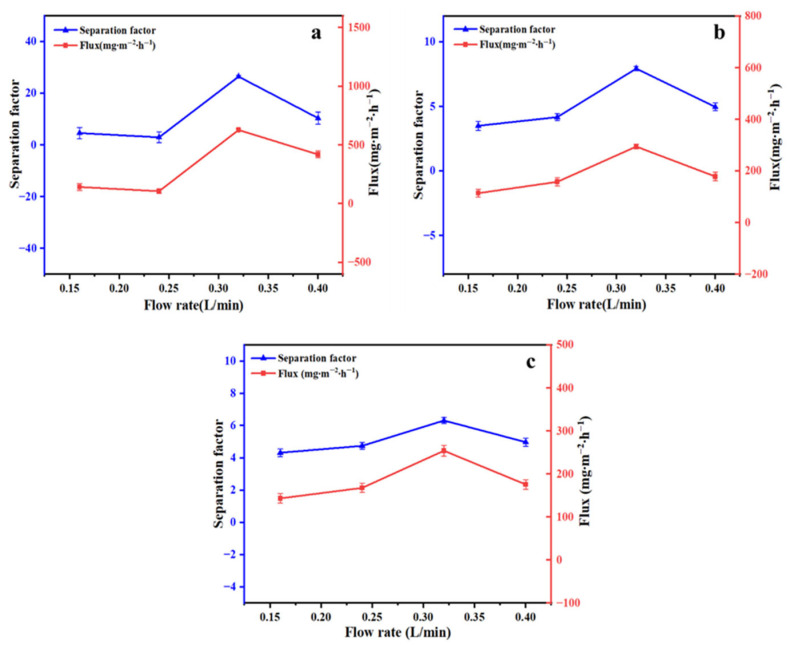
Pervaporation performance of the ZIF-67@PDMS composite membranes for linalool (**a**), benzaldehyde (**b**), and ethyl acetate (**c**) at different flow rates.

The effect of membrane surface flow rate on the separation performance of the membrane was investigated. Aqueous solutions of linalool, benzaldehyde, and ethyl acetate were prepared at a uniform concentration of 50 mg/L. At 50 °C, the flow rates were controlled at 0.16, 0.24, 0.32, and 0.40 L/min, and pervaporation was performed for 2.0 h. Figure 10 shows that the membrane surface flow rate influenced both the permeation flux and separation factor for the three aromatics. In the ZIF-67@PDMS membrane, the permeation flux and separation factor for linalool, benzaldehyde, and ethyl acetate first increased and then decreased with rising cross-flow rate, reaching their maxima at 0.32 L/min. This phenomenon was analyzed in concentration polarization and gel models: when flux was controlled by concentration polarization, increasing the flow rate enhanced membrane flux; however, excessively high flow rate reduced the pressure difference across the membrane, leading to a decline in flux [36].

### 3.4. Isolation of Aromatic Compounds from Strawberry Juice and Flavor Restoration of Concentrated Juice

The feasibility of applying pervaporation to restore the flavor of strawberry concentrate was investigated. Samples of freshly pressed strawberry juice (1#), PV retentate juice (2#), and strawberry concentrate fortified with PV permeate (3#) were taken for SPME-GC/MS analysis to compare the types and relative contents of aroma compounds among the three.

**Figure 11:**
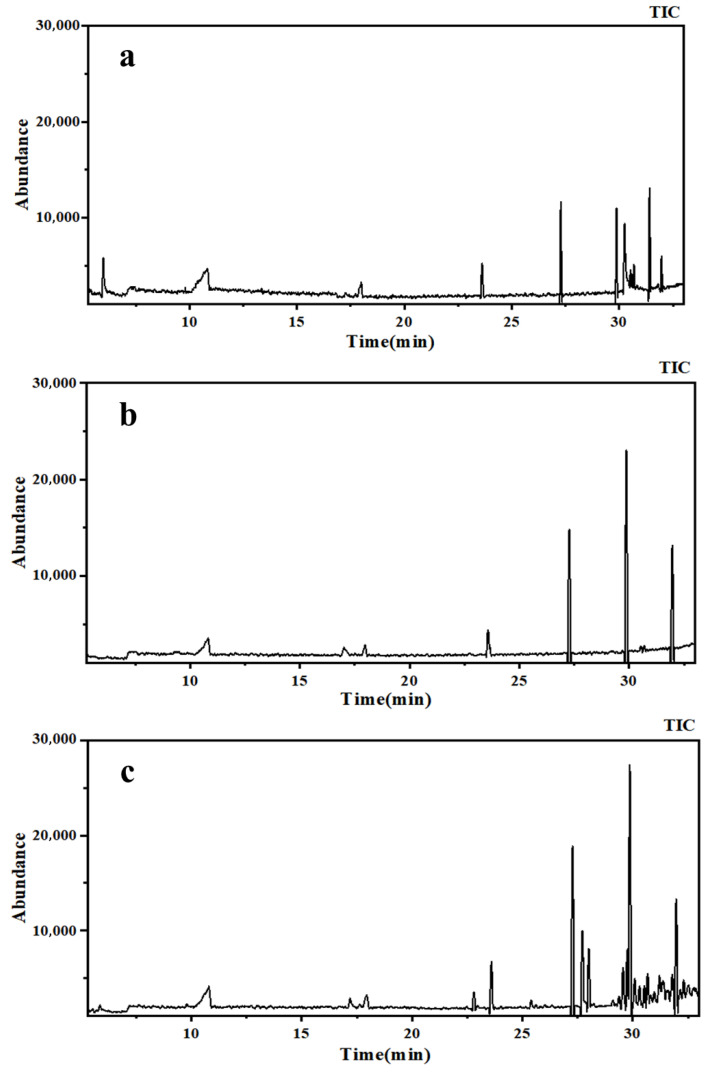
GC-MS total ion current chromatograms of volatile compounds in strawberry concentrate before (**a**) and after (**b**) pervaporation and following aroma replenishment (**c**).

As shown in Figure 11 and Table 1, the volatile constituents of Sample #1 were complex: 28 compounds were identified, comprising 9 esters, 5 ketones, 3 alcohols, 3 aldehydes, 3 alkanes, and 5 other types. In sample #2, 21 aroma compounds were detected, 17 of which were also present in #1 but at markedly lower abundances, confirming that PV effectively separated and recovered the aromatic fraction from the strawberry juice. Notably, the ketones present in Sample #1 were not detected in Sample #2, indicating that PDMS exhibits high separation selectivity for ketones [37]. This phenomenon may be attributed to the following synergistic mechanisms. (I) size screening effect: the molecular size or molecular weight cutoff value of ketones is just within the effective separation pore size of the membrane, which achieves separation with larger molecules (such as polysaccharides and proteins) or smaller molecules (such as water and organic acids). (II) specific interactions: on the one hand, the unsaturated cobalt (Co) sites exposed in ZIF-67, such as Lewis acid centers, have significant affinity for lone pair electrons on ketone carbonyls, and may achieve specific adsorption through weak coordination or dipole–dipole interaction [38]; on the other hand, there is a significant hydrophobic interaction between the strong hydrophobic surface of the PDMS matrix and the non-polar carbon chain in ketone molecules, which further prolongs the residence time of ketone molecules on the membrane surface and enhances their separation selectivity. Furthermore, four new compounds appeared in sample #2, which might have been due to several reasons. Firstly, the local thermal effect of the reaction site might have led to the decomposition of the target intermediate or triggered thermal catalytic side reactions such as dehydration, condensation, and C-C bond cleavage. Secondly, the specific active centers formed by the catalyst at the optimal loading level (such as metal sites in specific coordination environments) might have induced catalytic processes that deviated from the main reaction pathway. Thirdly, the active sites were abundant and well dispersed, which might have led to a local concentrated accumulation of intermediates on their surfaces, changed the reaction kinetics and thermodynamic equilibrium, and promoted the occurrence of side reactions [39,40]. A total of 25 compounds were identified in sample #3, including 3 alcohols, 8 esters, 3 ketones, 3 aldehydes, 3 alkanes, and 5 others—closely matching the profile of #1, their abundances were markedly higher than in #2, demonstrating that backfilling the PV permeate effectively restored both the composition and levels of compounds in the concentrated strawberry juice.

The results indicate that both our study and previous research have detected major or key compounds, such as terpenes, alcohols, esters, ketones, acids, and other volatile organic compounds [41]. Among these, esters (such as methyl esters, acetate esters, methyl phosphate esters, etc.) are the most abundant among all complex compounds and are an important component of strawberry fruit aroma, which is consistent with the research of Yao et al. [42]. Terpenoid compounds (such as linalool and nerol) impart strawberries with their delightful floral and fruity aroma [43], and fatty acid-derived aldehydes and ketones are the source of the green, fresh aromatic notes in strawberries [44]. In addition, in our study, we also found compounds that were not reported or had significantly different levels in other varieties or conditions. Of particular note, our analysis has, for the first time, detected the following compounds in the volatile components of strawberry fruit: methyl 1-[[(2-methylphenyl)imino]benzyloxy]-2-naphthoic acid, ethyl 4-isobutyryl-3,5-dimethyl-1H-pyrrole-2-carboxylate, and N-(2-phenylethyl)phenylethylamine. To our knowledge, these compounds have not been reported in the current published literature on strawberry fruit VOCs. Their detection may be attributable to differences in strawberry cultivars or growing conditions, or may relate to specific harvest times, processing methods, or storage practices.

### 3.5. Electronic Nose Analysis

**Figure 12:**
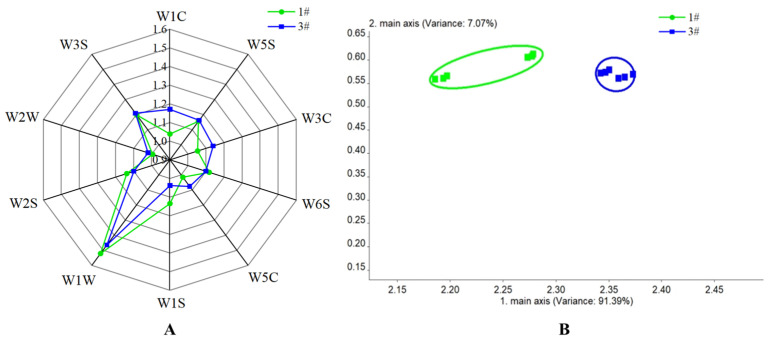
Electronic nose radar response plot (**A**) and PCA plot (**B**) of strawberry concentrate before pervaporation treatment and after aroma refilling.

To further verify the flavor restoration efficacy, an electronic nose was used to analyze the flavor characteristics of samples 1# and 3#. The electronic nose sensor was shown in Table 2. Figure 12A showed that the odor profiles of samples 1# and 3#were almost superimposed; sensor W1W gave the highest response, reflecting extreme sensitivity to sulfur compounds. This agreed with Liu et al. [45] and was probably attributable to naturally occurring strawberry thiols, sulfides or disulfides, or the reactions between certain special components in strawberries (such as fruit acids [46] and esters) and sulfides that generated volatile sulfur species and amplified the signal [47]. The figure showed that sample 3# gave stronger response on sensors W1C, W3C, and W5C than sample 1#. This was attributed to the membrane’s selective permeability and enrichment effect: certain aroma compounds preferentially permeated and accumulated in the permeate, these aroma components dominated in the refilled solution, as they were not interfered with by other substances from the original juice, making it easier for the aroma components to escape from the solution and be detected by the electronic nose sensors. To further distinguish the odor differences before and after pervaporation, PCA was performed on the electronic nose results. Figure 12B showed that PC1 accounted for 91.29% of the variance and PC2 for 7.07%, giving a cumulative 98.36%, indicated that this analysis can basically cover the main information of volatile components in the samples. The two samples scattered far apart in the score plot, indicating a pronounced difference in aroma composition between the original and reconstituted juices [48].

### 3.6. Research Limitations and Future Prospects

Although this study confirmed the excellent performance of pervaporation technology in the field of aroma separation and recovery of strawberry juice, in order to successfully promote its industrial application, the following challenges still need to be overcome: the presence of residual organic solvents, the long-term operation stability of the membrane materials, a systematic pollution prevention and control strategy, and a large-scale process design. Among these, the particle aggregation phenomenon and the service life of the membrane module in the actual operation are the key factors restricting the continuous and efficient separation performance of this technology. In the research into pervaporation for strawberry juice, the long-term operational stability and lifespan of zeolite membranes were constrained by multiple factors, primarily including operating conditions (such as temperature and pressure), the physicochemical properties of the feed solution system, and the structural integrity of the membrane material itself. Among these, cracks and defects potentially forming within the membrane layer represented one of the critical issues affecting zeolite membrane performance and longevity [49]. Such structural defects, typically induced by thermal stress, mechanical stress, or chemical degradation [50,51,52], not only shortened service life but also caused significant reductions in membrane selectivity and permeate flux. This led to undesirable permeation of non-target molecules, thereby diminishing overall separation efficiency [53]. Therefore, to effectively extend the service life of zeolite membranes, research into the optimization of zeolite membrane cleaning and regeneration strategies is the key problem to be solved in the next stage. Future research must establish a multi-level maintenance system from conventional physical and chemical cleaning to targeted deep regeneration treatments (such as in situ regeneration [35] and membrane surface modification regeneration), to ensure the sustainable recovery of membrane performance and long-term stable operation. Nevertheless, frequent cleaning and membrane lifespan limitations not only increase material consumption, energy expenditure, and downtime costs but also directly impact process economics. Consequently, large-scale applications need to further optimize the process design to ensure the continuous and stable operation of the industry.

In addition, the strategy proposed in this study shows significant potential for broad application across various juice systems. PDMS-based hydrophobic membranes have been demonstrated to preferentially permeate a range of hydrophobic volatile organic compounds (such as esters, terpenes, and aldehydes), which are the primary constituents of the characteristic aromas of most fruits [54,55]. This strategy relies on the membrane’s ability to separate “water–aroma mixtures” rather than targeting specific compounds, making it applicable for aroma separation in multiple fruit juices. Future research can optimize the membrane process’s parameters based on the material properties of different juices and evaluate its effectiveness in restoring the flavor profiles of specific juices.

## 4. Conclusions

Aroma is a critical determinant of food flavor. However, during food processing, particularly in the production of juice concentrates, a significant portion of volatile aroma compounds inevitably degrades or is lost through azeotropic entrainment, leading to a marked decline in the flavor quality of the final product.

To address this issue, we developed an effective strategy for the restoration of the aroma quality of strawberry concentrate, which comprises two steps, namely, the isolation of aromatic compounds from freshly squeezed juice using a ZIF-67@PDMS composite membrane, and the backfilling of the permeate to the concentrated strawberry juice. The experimental results showed that, owing to the inclusion of ZIF-67 particles, the ZIF-67@PDMS composite membrane exhibited significantly enhanced membrane performance compared to pure PDMS, ensuring the efficient separation of strawberry aromatics. Furthermore, SPME-GC/MS and electronic nose analysis demonstrated that both the variety and content of aromatic compounds in concentrated strawberry juice were notably restored by means of backfilling with permeate liquid, and the aroma profile of the restored sample was closely similar to that of fresh strawberry juice, indicating the effectiveness of the isolation–backfilling approach for restoring the aroma of strawberry concentrate. This two-step aroma restoration strategy is characterized by simplicity, low energy consumption, and high efficiency, rendering it a potentially promising and universal method for improving the flavor quality of concentrated juice.

## Figures and Tables

**Table 1:** Solid phase microextraction gas chromatography–mass spectrometry (SPME-GC/MS) was used to analyze fresh pressed strawberry juice (#1), PV retentate juice (#2), and strawberry concentrate fortified with PV permeate (#3) samples. The types and relative contents of aroma compounds among the three were compared, and the recovery rate of aroma compounds was calculated.

No.	R_T_	Compounds	RIexp	RI Lit	Peak Area, mV∙s			Recovery Rate (%)
					#1	#2	#3	
1	32.41	Nerolidol	1522	1528	13,010	2481	4484	80.93
2	10.90	phenethyl alcohol	1109	1116	3210	2212	2030	31.09
3	10.82	Linalool	1088	1098	4710	2425	3827	48.51
4	41.50	3-(5-Mercapto-1,3,4-oxadiazol-2-yl)phenol	1682	-	7530	3039	4734	59.64
5	39.53	Di(1-phenylpropyl) isophthalate	1652	-	1127	-	549	-
6	45.39	Acetic acid 3-(4-chloro-phenyl)-1,4-dioxo-1,4-dihydro-naphthalen-2-yl ester	1721	-	1124	-	525	-
7	38.94	4-hexen-1-ol acetate	1635	-	1668	-	-	-
8	5.99	Ethyl acetate	633	645	5822	1503	1665	74.18
9	46.31	Dicyclohexyl methyl phosphonate	1741	-	6245	1754	2910	71.91
10	58.80	2-Thiopheneacetic acid but-3-yn-2-yl ester	1834	-	5270	3011	4189	42.86
11	40.64	Ethyl 4-isobutyryl-3,5-dimethyl-1H-pyrrole-2-carboxylate	1669	-	3051	-	1058	-
12	41.87	1-[[(2-methylphenyl)imino]benzyloxy]-2-naphthoic acid methyl ester	1688	-	11,458	3029	6019	73.56
13	44.75	[2,2′-Bifuran]-3-carboxaldehyde methyl ester	1713	-	7877	2028	3735	74.25
14	30.98	1-Benzyl-1H-indole-3-carbaldehyde	1383	-	2762	2364	3454	14.41
15	30.67	1H-1,2,3-triazole-4-carbaldehyde	1367	-	4847	2696	5450	44.38
16	45.81	2′-Methyl acetophenone	1728	-	892	-	-	-
17	38.65	4-Methylphenyl pentanone	1626	-	965	-	-	-
18	9.80	benzaldehyde	946	961	3608	1945	2235	46.09
19	30.22	2-Hydroxy-4,5-methylenedioxyacetophenone	1360	-	7742	-	2192	-
20	41.43	3-(3,3-Dimethylbutyl)cyclohexanone	1678	-	2537	-	2648	-
21	40.77	2-Phenyl-1H-1,2,4-triazol-5-one	1673	-	2483	-	1548	-
22	20.07	1-(5′-methylfurfuryl)pyrrolidine	1225	-	1800	-	1217	-
23	23.64	hexafluoroethane	1267	-	3993	2466	3584	38.24
24	43.75	1-Cyclohexyl dimethyl siloxy-3-phenyl propane	1701	-	5667	3487	4598	38.47
25	45.89	1-(2,2,2-Trifluoroethyl)-1H-pyrazol-3-amine	1732	-	1699	-	3222	-
26	44.68	4′-Pentyl-[1,1′-biphenyl]-4-carbonitrile	1710	-	3386	2470	2905	27.05
27	52.32	5-phenylthiazole	1783	-	5864	536	-	90.86
28	27.25	2-methylnaphthalene	1311	1296	-	-	6981	-
29	23.62	4-(1-Methylcyclobutyl)phenol	1263	-	-	3242	-	-
30	30.69	5-hexyl-2,4-dihydroxybenzaldehyde	1370	-	-	1438	-	-
31	49.01	4-Nitro-1H-isoindole-1,3(2H)-dione	1759	-	-	387	-	-
32	49.51	2-Acetyl-3-nitrobenzoic acid	1768	-	-	3012	-	-
33	40.47	N-(2-phenylethyl)phenylethylamine	1661	-	9029	2393	2950	73.50

R_T_ = retention time. RIexp = retention index experimental. RI lit = retention index literature database.

**Table 2:** Sensitive substances the PEN3 electronic nose sensor.

Serial Number	Sensor Name	Sensitive Substances
1	W1C	Aromatic compounds
2	W5S	Nitrogen oxides
3	W3C	Ammonia, aromatic molecules
4	W6S	Hydrides
5	W5C	Olefins, aromatic, polar molecules
6	W1S	Alkanes
7	W1W	Sulfur compounds
8	W2S	Alcohols, some aromatic compounds
9	W2W	Aromatic compounds, sulfur-containing organic compounds
10	W3S	Alkanes and fatty acids

## Data Availability

The original contributions presented in this study are included in the article. Further inquiries can be directed to the corresponding author.
